# Silicon Regulates Potential Genes Involved in Major Physiological Processes in Plants to Combat Stress

**DOI:** 10.3389/fpls.2017.01346

**Published:** 2017-08-03

**Authors:** Abinaya Manivannan, Yul-Kuyn Ahn

**Affiliations:** ^1^Vegetable Research Division, National Institute of Horticultural and Herbal Science, Rural Development Administration Jeonju, South Korea; ^2^Department of Vegetable Crops, Korea National College of Agriculture and Fisheries Jeonju, South Korea

**Keywords:** defense response, gene regulation, photosynthesis, polyamine biosynthesis, regulatory elements

## Abstract

Silicon (Si), the quasi-essential element occurs as the second most abundant element in the earth's crust. Biological importance of Si in plant kingdom has become inevitable particularly under stressed environment. In general, plants are classified as high, medium, and low silicon accumulators based on the ability of roots to absorb Si. The uptake of Si directly influence the positive effects attributed to the plant but Si supplementation proves to mitigate stress and recover plant growth even in low accumulating plants like tomato. The application of Si in soil as well as soil-less cultivation systems have resulted in the enhancement of quantitative and qualitative traits of plants even under stressed environment. Silicon possesses several mechanisms to regulate the physiological, biochemical, and antioxidant metabolism in plants to combat abiotic and biotic stresses. Nevertheless, very few reports are available on the aspect of Si-mediated molecular regulation of genes with potential role in stress tolerance. The recent advancements in the era of genomics and transcriptomics have opened an avenue for the determination of molecular rationale associated with the Si amendment to the stress alleviation in plants. Therefore, the present endeavor has attempted to describe the recent discoveries related to the regulation of vital genes involved in photosynthesis, transcription regulation, defense, water transport, polyamine synthesis, and housekeeping genes during abiotic and biotic stress alleviation by Si. Furthermore, an overview of Si-mediated modulation of multiple genes involved in stress response pathways such as phenylpropanoid pathway, jasmonic acid pathway, ABA-dependent or independent regulatory pathway have been discussed in this review.

## Introduction

The surface of earth is covered with 27.70% of silicon (Si) next to oxygen, but the existence of Si in its pure form is rare (Mitra, 2015). Silicon is deposited in the form of quartz (SiO_2_), sand, and sand stone in the earth crust (Rédei, 2008). In biological organisms, Si occurs in the form of amorphous silica (SiO_2_ nH_2_O) and soluble silicic acid (Si(OH)_4_) (Das and Chattopadhyay, 2000). Moreover in eukaryotes, Si is important for bones, cartilage, connective tissue formation, enzymatic activities, and lymphocyte proliferation (Carlisle, 1988, 1997). In plants, Si is absorbed as an uncharged monomeric silicic acid in the pH range below 9 (Knight and Kinrade, 2001; Ma and Yamaji, 2006). The level of Si accumulation by plants can be directly correlated with the beneficial effects attributed by Si. Among the plants, monocots like rice, sugarcane, maize, and cereals absorb Si in large quantities on comparison with dicots due to the presence of Si transporters (Ma et al., 2016). The absorption and transportation of Si in plants is a complex process which involves influx and efflux Si transporters belonging to aquaporin family with specific selectivity properties. For instance, the high Si accumulator like rice consists of low silicon rice 1 (Lsi1) transporter, a nodulin 26-like intrinsic protein (NIP) in roots.

Recently, several putative silicon transporters have been identified in monocot and dicot plants by Deshmukh et al. (2015). According to the report, uptake of Si is particularly confined to the plant species consisting of NIP type aquaporins with GSGR selectivity filter along with an exact distance of 108 amino acids between the asparagine-proline-alanine (NPA) domain (Deshmukh et al., 2015). The exogenous supplementation of Si proves to be beneficial for plants particularly under abiotic and biotic stress conditions (Supplementary Table 1). Silicon nutrition resulted in the improvement of growth and development (Eneji et al., 2008; Soundararajan et al., 2014; Zhang et al., 2015), increase in yield (Epstein, 1999), abiotic and biotic stress tolerance (Ma, 2004; Zhu et al., 2004; Liang et al., 2007; Muneer et al., 2014), management of macro and micro nutrients (Tripathi et al., 2014), resistance against pest and pathogens (Lanning, 1966; Cookson et al., 2007).

Apart from the abovementioned advantages, Si augmentation in soil-less cultivation of corn salad improved the edible yield, quality, and shelf life of baby leaf vegetable corn salad by the regulation of nutrient acquisition, uptake of nitrate/iron, phenoloxidase gene expression, and protection of chlorophyll degradation (Gottardi et al., 2012). Likewise, Si inclusion in tissue culture medium resulted in the enhancement of axillary shoot induction (Manivannan et al., 2017), alleviation of hyperhydricity (Soundararajan et al., 2017a), callus induction (Islam et al., 2005), and root morphogenesis (Asmar et al., 2013). Even though, the effect of Si in plants was studied for several years, the mechanisms behind the physiological responses or molecular regulation in plants upon Si nutrition under normal and stressed conditions is still under study.

Broadly, Si-mediated tolerance to stress can be interpreted either in the form of mechanical barrier through Si(OH)_4_ polymerization in cell walls to prevent the penetration of host tissue by pest or pathogen (Yoshida et al., 1962) or by triggering the chemical resistance mechanism (Fawe et al., 1998). According to Chérif et al. (1992), in cucumber the Si treatment increased the activities of chitinases, peroxidases, and polyphenoloxidases against *Pythium ultimum*. Similarly, Si nutrition enhanced the plant growth by the regulation of antioxidant and nutrient uptake in salt stressed in Salvia (Soundararajan et al., 2014). Moreover, Si retarded the Na^+^ and Cl^−^ transportation due to silicon deposition to cope up the plants under salinity stress (Gong et al., 2006; Shi et al., 2013). Likewise, Si supplementation decreased metal toxicity such as toxicity of aluminum (Al) (Wang et al., 2004), boron (B) (Gunes et al., 2007), cadmium (Cd) (Liang et al., 2005), chromium (Cr) (Tripathi et al., 2012) copper (Cu) (Li et al., 2008), and zinc (Zn) (Neumann and Zur Nieden, 2001). Recently Debona et al. (2017), has elaborately reviewed the possible stress tolerance mechanisms attributed by Si upon abiotic and biotic stresses. According to the review, upon metal toxicity, silicon tends to modulate the pH range of soil, changes the metal speciation, compartmentalization and co-precipitation of metals, and sequestration strategies to combat the metal stress (Debona et al., 2017). In addition, the Si-fortified fertilizers are gaining interest in recent days due to its beneficial results particularly in the improvement of growth, photosynthesis, and maintenance of electrolyte leakage even under stressed conditions (Chen et al., 2011).

Overall, the inclusion of Si is important for plant growth and numerous reports and reviews illustrated the Si dependent modulations of antioxidant enzymes, nutrient contents, homeostasis in reactive oxygen species however, very few studies have dealt with the Si-mediated molecular regulation of genes in plants under abiotic and biotic stresses (Brunings et al., 2009; Song et al., 2014; Yin et al., 2016). The modern high-throughput approaches can aid in deciphering the important genes involved in the Si-mediated stress response in plants (Tables 1–3). The Si-dependent expression of genes was first investigated in rice using the microarray approach by Watanabe et al. (2004). According to the results, the addition of Si up-regulated the abundance of a zinc finger protein homolog and down-regulated the expressions of chlorophyll *a*/*b* binding protein, metallothione-like protein, *Xa21* gene family member, and carbonic anhydrase homolog (Watanabe et al., 2004). In general, the zinc finger proteins act as the major transcription factors for stress responsible genes and the enhancement of its expression can increase the regulation of stress responsible genes which might increase the stress tolerance in Si treated plants (Watanabe et al., 2004). In the following sections, the Si-mediated regulations of genes involved in several physiological processes have been discussed.

**Table 1:** List of genes up regulated upon the supplementation of Si under abiotic stress.

**Abiotic stress**	**Gene identifier**	**Functional annotation**	**Process**	**Organism**	**References**
Metal toxicity	*Os08g02630*	Subunit of oxygen evolving complex-PSII	Photosynthesis	*Oryza sativa*	Song et al., 2014
Metal toxicity	*Os05g48630*	Photosynthetic co8y stability maintenance	Photosynthesis	*Oryza sativa*	Song et al., 2014
Metal toxicity	*Os07g37030*	Maintenance of cytochrome	Photosynthesis	*Oryza sativa*	Song et al., 2014
Metal toxicity	*Os03g57120*	Ferrodoxin NADP^+^ reductase	Photosynthesis	*Oryza sativa*	Song et al., 2014
Metal toxicity	*Os09g26810*	Subunit of LHC II complex	Photosynthesis	*Oryza sativa*	Song et al., 2014
Metal toxicity	*Os04g38410*	Subunit of LHC II complex	Photosynthesis	*Oryza sativa*	Song et al., 2014
Drought	*AK070732*	Member of RING domain containing protein family	Regulatory gene	*Oryza sativa*	Khattab et al., 2014
Drought	*AF300971*	Dehydration responsive element binding protein	Regulatory gene	*Oryza sativa*	Khattab et al., 2014
Drought	*AJ578494*	Choline monooxygenase	Regulatory gene	*Oryza sativa*	Khattab et al., 2014
Drought	*AB028184*	NAC regulons (No apical meristem(NAM), *Arabidopsis thaliana* activating factor [ATAF], and cup-shaped cotyledon [CUC])	Regulatory gene	*Oryza sativa*	Khattab et al., 2014
Drought	*NM_001074375*	Dehydrin	Regulatory gene	*Oryza sativa*	Khattab et al., 2014
Salt stress	*Sb02g025110*	S-Adenosyl-L-methionine decarboxylase	Polyamine synthesis	*Sorghum bicolor*	Yin et al., 2016
Salt stress	*Sb04g025720*	S-Adenosyl-Metdecarboxylase	Polyamine synthesis	*Sorghum bicolor*	Yin et al., 2016
Salt stress	*Sb06g021540*	S-Adenosyl-Metdecarboxylase	Polyamine synthesis	*Sorghum bicolor*	Yin et al., 2016
Salt stress	*Sb10g002070*	Arginine decarboxylase	Polyamine synthesis	*Sorghum bicolor*	Yin et al., 2016
Salt stress	*Sb04g021790*	N-Carbamoyl putrescine amidohydrolase	Polyamine synthesis	*Sorghum bicolor*	Yin et al., 2016
Metal toxicity	*At5g22460*	Esterase lipase thioesterase family protein	Transporter gene	*Arabidopsis thaliana*	Li et al., 2008
Metal toxicity	*At5g59030*	Copper transporter	Transporter gene	*Arabidopsis thaliana*	Li et al., 2008

**Table 2:** List of genes up regulated upon the supplementation of Si under biotic stress.

**Biotic stress**	**Gene identifier**	**Functional annotation**	**Biological process**	**Organism**	**References**
Rice blast disease	*Os01g0713200*	β-1,3-Glucanase precursor	Defense	*Oryza sativa*	Brunings et al., 2009
Rice blast disease	*Os02g0584800*.	Heavy metal transport/detoxification protein domain-containing protein	Defense	*Oryza sativa*	Brunings et al., 2009
Rice blast disease	*Os02g0585100*	Heavy metal transport/detoxification protein domain-containing protein	Defense	*Oryza sativa*	Brunings et al., 2009
Rice blast disease	*Os04g0469000*	Heavy metal transport/detoxification protein domain-containing protein	Defense	*Oryza sativa*	Brunings et al., 2009
Rice blast disease	*Os04g0610400*	Pathogenesis-related transcriptional factor and ERF domain-containing protein	Defense	*Oryza sativa*	Brunings et al., 2009
Rice blast disease	*Os07g0104100*	Peroxidase precursor	Defense	*Oryza sativa*	Brunings et al., 2009
Rice blast disease	*Os11g0692500*	Bacterial blight resistance Protein	Defense	*Oryza sativa*	Brunings et al., 2009
Rice blast disease	*Os01g0963200*	Peroxidase BP 1 precursor	Defense	*Oryza sativa*	Brunings et al., 2009
Rice blast disease	*Os01g0378100*	Peroxidase precursor	Defense	*Oryza sativa*	Brunings et al., 2009
Bacterial wilt	*AF494201*	Tomato stress-responsive factor	Defense	*Solanum lycopersicum*	Ghareeb et al., 2011b
Bacterial wilt	*M69247*	Pathogenesis-related protein 1	Defense	*Solanum lycopersicum*	Ghareeb et al., 2011b
Bacterial wilt	*M80604*	β-Glucanase	Defense	*Solanum lycopersicum*	Ghareeb et al., 2011b
Bacterial wilt	*U30465*	Chitinase class II	Defense	*Solanum lycopersicum*	Ghareeb et al., 2011b
Bacterial wilt	*X94943*	Peroxidase	Defense	*Solanum lycopersicum*	Ghareeb et al., 2011b
Bacterial wilt	*M83314*	Phenylalanine ammonia lyase	Defense	*Solanum lycopersicum*	Ghareeb et al., 2011b
Bacterial wilt	*X99147*	Arabinogalactan protein	Defense	*Solanum lycopersicum*	Ghareeb et al., 2011b
Bacterial wilt	*L26529*	Polygalacturonase inhibitor protein	Defense	*Solanum lycopersicum*	Ghareeb et al., 2011b
Rice blast disease	*Os02g0807000*	Phosphoenolpyruvate carboxylase kinase	Housekeeping gene	*Oryza sativa*	Brunings et al., 2009
Rice blast disease	*Os01g0554100*	RNA-directed DNA polymerase (reverse transcriptase) domain containing protein	Housekeeping gene	*Oryza sativa*	Brunings et al., 2009
Rice blast disease	*Os03g0803500*	2OG-Fe(II) oxygenase domain-containing protein	Housekeeping gene	*Oryza sativa*	Brunings et al., 2009
Rice blast disease	*Os10g0559500*	2OG-Fe(II) oxygenase domain-containing protein	Housekeeping gene	*Oryza sativa*	Brunings et al., 2009
Rice blast disease	*Os09g0432300*	AAA ATPase, central region domain-containing protein	Housekeeping gene	*Oryza sativa*	Brunings et al., 2009
Rice blast disease	*Os06g0676700*	High pI α-glucosidase	Housekeeping gene	*Oryza sativa*	Brunings et al., 2009
Rice blast disease	*Os08g0190100*	Oxalate oxidase-like protein	Housekeeping gene	*Oryza sativa*	Brunings et al., 2009
Rice blast disease	*Os05g0495600*	P-type ATPase	Housekeeping gene	*Oryza sativa*	Brunings et al., 2009
Rice blast disease	*Os03g0405500*	PDI-like protein	Housekeeping gene	*Oryza sativa*	Brunings et al., 2009
Bacterial wilt	*AY157064*	WRKY group II transcription Factor	Regulatory gene	*Solanum lycopersicum*	Ghareeb et al., 2011b
Bacterial wilt	*AY383630*	Jasmonate and ethylene responsive factor 3	Regulatory gene	*Solanum lycopersicum*	Ghareeb et al., 2011b
Bacterial wilt	*Z75520*	Ferredoxin I	Photosynthesis	*Solanum lycopersicum*	Ghareeb et al., 2011b

**Table 3:** List of genes down regulated upon the supplementation of Si under abiotic and biotic stresses.

**Stress**	**Gene identifier**	**Functional annotation**	**Biological process**	**Organism**	**References**
Rice blast disease	*Os11g0608300*	Barley stem rust resistance protein	Defense	*Oryza sativa*	Brunings et al., 2009
Rice blast disease	*Os11g0673600*	Disease resistance protein family protein	Defense	*Oryza sativa*	Brunings et al., 2009
Rice blast disease	*Os03g0266300*	Heat shock protein Hsp20 domain-containing protein	Defense	*Oryza sativa*	Brunings et al., 2009
Rice blast disease	*Os03g0235000*	Peroxidase	Defense	*Oryza sativa*	Brunings et al., 2009
Rice blast disease	*Os12g0491800*	Terpene synthase-like domain-containing protein	Defense	*Oryza sativa*	Brunings et al., 2009
Rice blast disease	*Os10g0191300*	Type 1 pathogenesis-related protein	Defense	*Oryza sativa*	Brunings et al., 2009
Rice blast disease	*Os09g0417800*	DNA-binding WRKY domain-containing protein	Regulatory gene	*Oryza sativa*	Brunings et al., 2009
Rice blast disease	*Os08g0332700*	Trans-acting transcriptional protein ICP0	Regulatory gene	*Oryza sativa*	Brunings et al., 2009
Rice blast disease	*Os02g0695200*	P-type R2R3 Myb protein	Regulatory gene	*Oryza sativa*	Brunings et al., 2009
Rice blast disease	*Os09g0110300*	Putative cyclase family protein	Housekeeping gene	*Oryza sativa*	Brunings et al., 2009
Rice blast disease	*Os08g0112300*	Transferase family protein	Housekeeping gene	*Oryza sativa*	Brunings et al., 2009
Rice blast disease	*Os10g0154700*	Cyclophilin Dicyp-2	Housekeeping gene	*Oryza sativa*	Brunings et al., 2009
Rice blast disease	*Os08g0155700*	DNA-directed RNA polymerase largest chain (isoform B1)-like protein	Housekeeping gene	*Oryza sativa*	Brunings et al., 2009
Rice blast disease	*Os11g0194800*	DNA-directed RNA polymerase II	Housekeeping gene	*Oryza sativa*	Brunings et al., 2009
Rice blast disease	*Os11g0106700*	Ferritin 1, chloroplast precursor	Housekeeping gene	*Oryza sativa*	Brunings et al., 2009
Rice blast disease	*Os12g0258700*	Multi copper oxidase, type 1 domain-containing protein	Housekeeping gene	*Oryza sativa*	Brunings et al., 2009
Rice blast disease	*Os01g0770200*	Tyrosine decarboxylase 1	Housekeeping gene	*Oryza sativa*	Brunings et al., 2009
Rice blast disease	*Os01g0627800*	Cytochrome P450 monooxygenase	Photosynthesis	*Oryza sativa*	Brunings et al., 2009
Salt stress	*Sb01g009450*	1-Aminocyclopropane-1-carboxylic acid synthase	Polyamine synthesis	*Sorghum bicolor*	Yin et al., 2016

## Silicon regulated the genes involved in photosynthesis upon metal toxicity

Among several mechanism of Si-mediated stress amelioration, the primary stress-combating strategies utilized by Si is the enhancement of photosynthesis process in the stressed plants. Broadly, the oxidative stress resulting from both abiotic and biotic stress target photosynthesis by affecting the major enzymes in calvin cycle and photosynthetic electron transport chain (Nwugo and Huerta, 2008; Gong and Chen, 2012; Muneer et al., 2014). Even though, various studies have evidenced the beneficial effects of Si on photosynthesis, only a few have examined the molecular rationale behind the gene expression upon Si addition, particularly in rice. The report by Song et al. (2014) illustrated the transcriptional regulation of photosynthesis related genes under Si amendment and zinc stress. Supplementation of Si increased the transcript levels of *PsbY* (Os08g02630), a vital polyprotein involved in photosystem II (PSII) whereas, the Zn in higher concentration retarded the *PsbY* expression. In detail, the *PsbY* is a subunit of oxygen-evolving complex of PSII with manganese-binding polypeptide consisting L-arginine metabolizing enzyme activity (Kawakami et al., 2007). Furthermore, the Si-mediated increase in the level of *PsbY* transcripts could activate the manganese-binding capacity, oxidation of water that might improve the efficiency of PS II and electron transfer rate (Song et al., 2014). Likewise, the application of Si has improved the abundance of *PsaH* which encodes the vital polypeptide subunits in the PSI dimer (Pfannschmidt and Yang, 2012). The *PsaH* knockout mutant damaged the LCH-II complex resulting in the energy transition delay between PS II and PS I (Lunde et al., 2000).

**Figure 1:**
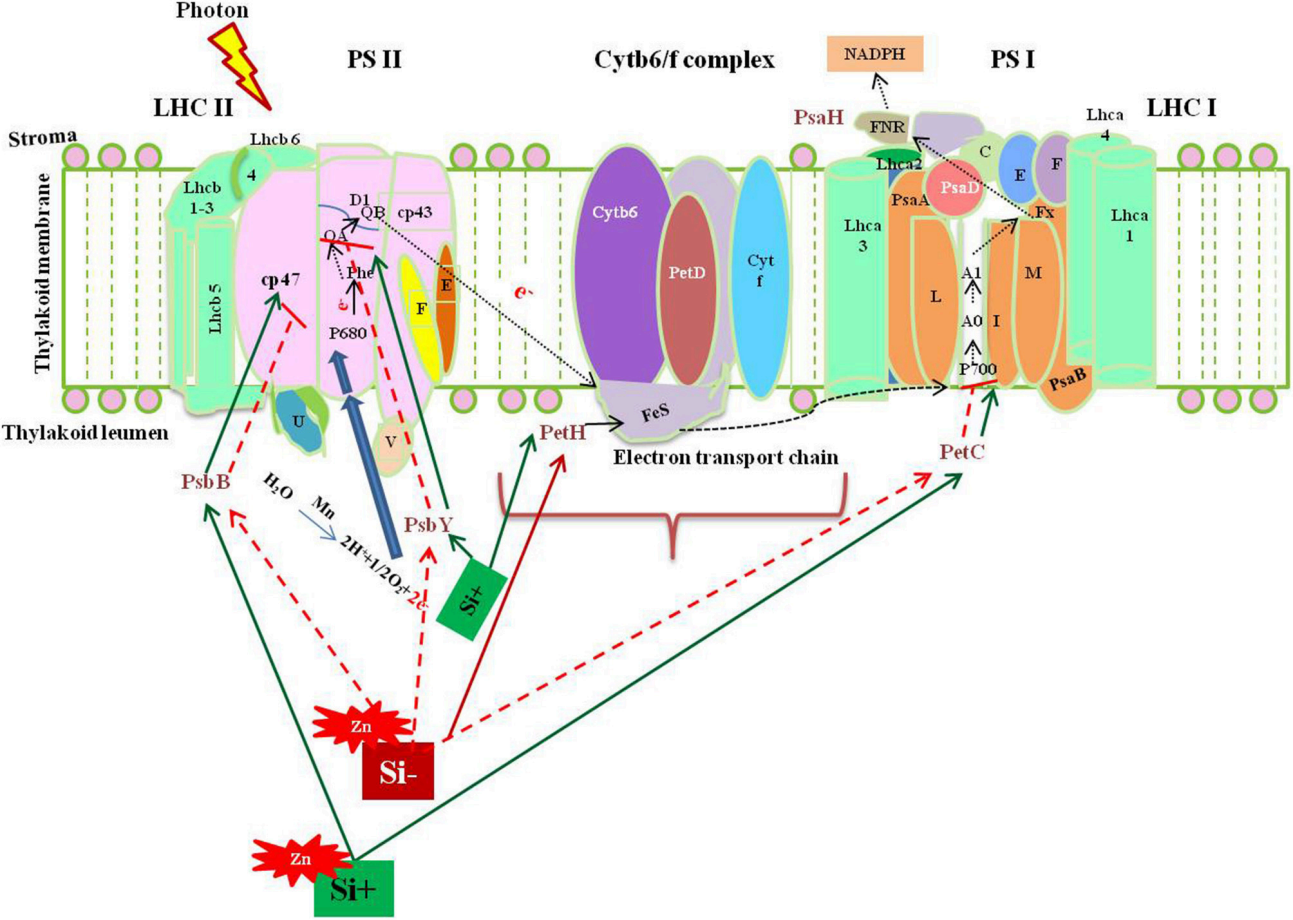
A putative model representing the regulation of photosynthesis related genes upon metal stress with or without Si supplementation. The green standard arrows represent the up-regulation of genes progressing with normal function in the presence of Si, blue standard arrows represent the entry of electrons into the PS II, flow of electron in the photosynthetic cascade has been represented by dotted black arrows, and red dotted arrows indicate the down-regulation of photosynthetic genes and corresponding gene functions upon metal stress in Si- plant. PSII, Photosystem II; Cytb6/f, cytochrome b6/f complex; PSI, photosystem 1; LHC I, light harvesting complex I. The diagram was conceived based on the interpretation from the following literatures (Lunde et al., 2000; Kawakami et al., 2007; Song et al., 2014).

Similarly, the Zn toxicity resulted in the down regulation of *PetC* which has been recovered by Si supplementation. In general, *PetC* codes Rieske Fe-S center-binding polypeptide of cytochrome *bf* complex which is responsible for the proper functioning of cytochrome (Breyton et al., 1994). Hence, the Si mediated up-regulation of *PetC* could augment the structural integrity of the chloroplast (Song et al., 2014). Moreover, Si treatment increased the expression of *PetH* in similar manner with *PetC*. The product of *PetH* is ferredoxin NADP+ reductase, an important enzyme involved in the synthesis of NADPH via photosynthetic electron transport chain. Furthermore, reducible glutathione content in the cells is maintained by *PetH* (Song et al., 2014). In addition to the above listed genes, the supplementation of Si resulted in the up-regulation of genes (*Os03g57120* and *Os09g26810*) involved in the light harvesting complex. Thus, the molecular insight into Si-dependent up-regulation of genes associated with PS I and PS II illustrate the positive effects rendered by Si on photosynthesis process. The physiological improvement of photosynthetic apparatus and reduction in the degradation of chlorophyll pigmentation reported by several researches can be correlated with the genic regulation of photosynthetic genes by Si at molecular level. A putative model representing the Si-mediated regulation of photosynthesis associated genes discussed above have been illustrated in Figure 1. Overall, the augmentation of Si instigated the expression levels of important genes in both photosystems to increase the efficiency of photosynthesis particularly under stressful environment.

## Silicon modulated the expression of housekeeping genes upon pathogen infection

In general, housekeeping genes are expressed constitutively in all cells regardless of its patho-physiological state and these genes are vital for the maintenance of proper functioning of cells. Although, the expression of housekeeping genes is constant, several studies illustrated their loss of stability under stressed conditions (Nicot et al., 2005; Jain et al., 2006). According to Brunings et al. (2009), the supplementation of Si down regulated the expression of important housekeeping genes in rice under normal condition however, upon pathogen infection Si up-regulated the housekeeping genes to maintain the cellular functions. Similarly, Ghareeb et al. (2011a) observed the Si-mediated up-regulation of housekeeping genes such as actin (*ACT*), alpha-tubulin (*TUB*), and phosphoglycerate kinase (*PGK*) in *Ralstonia solanacearum* infected tomato. According to Jarosch et al. (2005) actin cytoskeleton provided the basal resistance against the *R. solanacearum*. Therefore, the Si dependent up-regulation of actin in tomato plants induced the host resistance (Ghareeb et al., 2011a). Tomato is considered as the low-level silicon accumulator (~0.2% dry weight) because of the lack of high density Si transporter (Ma and Yamaji, 2006). Moreover, the impermeability of Si by nodulin 26-like intrinsic protein (NIP) in tomato (SINIP2-1) has been postulated due to the difference in the spacing between two NPA domains which forms the half helix inserts in SINIP2-1. However, the meager uptake of Si by low silicon accumulating plants is unclear but it might be possible that the lesser uptake of Si by tomato plants particularly under stressed environment might be due to the existence of a passive uptake mechanism. Moreover, the application of Si even in less biological concentration in the low accumulating species such as tomato (Romero-Aranda et al., 2006), capsicum (Jayawardana et al., 2015), and roses (Soundararajan et al., 2017b) has rendered abiotic and biotic stress tolerance. Despite the constant expression nature of housekeeping genes, variation in the expression levels upon Si amendment and pathogen infection could induce the basal defense mechanism in the host plant to protect from the pathogen. Taken together, the silicon amendment regulated the expression of vital housekeeping genes to alleviate the biotic stress.

## Silicon altered the expression of regulatory elements associated with stress response genes

**Figure 2:**
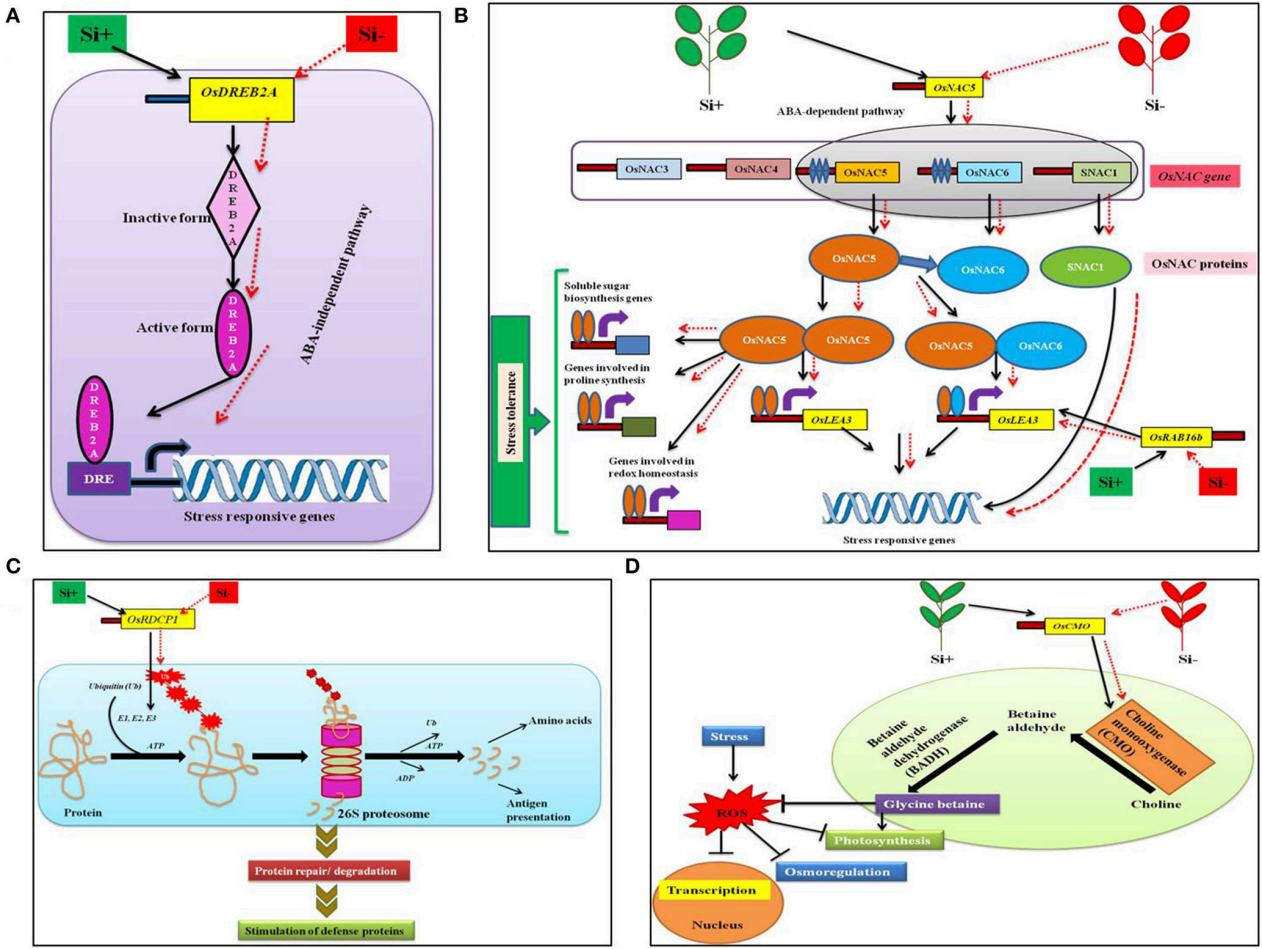
A schematic representation of the regulation of transcription factors under abiotic stress condition with or without Si supplementation. **(A)** Model displaying the *OsDREB2A* regulation in ABA-independent pathway to combat stress. **(B)** Regulation of *OsNAC5* transcription factor in ABA-dependent pathway to trigger stress tolerance related genes. **(C)** The *OsRDCP1* mediated stress tolerance response via the ubiquitin-proteosome degradation pathway. **(D)** Improvement of glycine betaine biosynthesis by *OsCMO* to combat ROS generation. The black standard arrows represent the up-regulation of gene in the presence of Si and red dotted arrows indicate the down-regulation of gene and corresponding functions upon stress in Si- plant. DRE, Dehydration responsive element; DREB2A; dehydration-responsive element binding protein 2A; NAC, no apical meristem (NAM), *Arabidopsis thaliana* activating factor (ATAF), and cup-shaped cotyledon (CUC) regulons; *OsRDCP1, Oryza sativa RING* domain containing protein; *OsCMO, Oryza sativa* choline monooxygenase; *SNAC1*, stress-responsive NAC protein, OsLEA3, *Oryza sativa* late embryogenesis abundant protein; E1, ubiquitin activating; E2, ubiquitin conjugating; E3,ubiquitin ligating enzymes, ATP, adenosine triphosphate; ADP, adenosine diphosphate. The diagram was conceived based on the interpretation from the following literatures (Mizoi et al., 2012; Nakashima et al., 2012; Khattab et al., 2014).

Stressful environment can induce the expression of myriads of genes involved in stress tolerance, metabolic processes, and signal transduction, etc. in plants (Shinozaki and Yamaguchi-Shinozaki, 2000; Xiong et al., 2002; Rabbani et al., 2003; Shinozaki et al., 2003). Amongst the stress induced genes, transcription factors (TF) are the primary regulators of the downstream genes important for plant tolerance against biotic and abiotic stresses (Gao et al., 2007; Lucas et al., 2011). In general, TFs are facilitated by particular cis-elements called regulons that are located in the promoter section of the target gene (Nakashima et al., 2009; Qin et al., 2011). Broadly, plants consists of a diverse number of regulons responding to stress, for example dehydration-responsive element binding protein (*DREB2*) are triggered by temperature and drought stress (Mizoi et al., 2012). Similarly, the *NAC* regulons [no apical meristem (NAM), *Arabidopsis thaliana* activating factor (ATAF), and cup-shaped cotyledon (CUC)] can be activated by osmotic stress in plants (Nakashima et al., 2009; Fujita et al., 2011). Moreover, the increase in the expression levels of TFs can stimulate a wide range of signal transduction pathways resulting in stress tolerance (Chaves and Oliveira, 2004; Umezawa et al., 2006). According to Khattab et al. (2014), in rice the addition of Si resulted in the up-regulation of TFs involved in the expression of *DREB2A, NAC5, Oryza sativa RING* domain containing protein *(OsRDCP1), Oryza sativa* choline monooxygenase (*OsCMO*), and dehydrin *OsRAB16b* (Figure 2). In rice, the *OsDREB* triggers the expression of stress-responsive genes that impart tolerance against osmotic stress in abscisic acid (ABA)—independent manner (Figure 2A) (Dubouzet et al., 2003; Hussain et al., 2011). In addition, the elevated levels of *OsDREB2A* provided drought resistance in rice (Chen et al., 2008; Wang et al., 2008). Similarly, NACs are TFs with various roles in development and stress response of plants (Tran et al., 2010). According to Fang et al., the rice genome consists of ~140 putative *NAC* or *NAC*-like genes among them 20 genes including *OsNAC5* are classified as stress responsive genes involved detoxification, redox homeostasis, and macromolecule fortification (Hu et al., 2008). Hence, the Si-mediated enhancement of *OsNAC5* transcripts led to prevention of lipid peroxidation and generation of excess hydrogen peroxide (H_2_O_2_). The abovementioned metabolic modulations shield the plants from dehydration and oxidative damages caused in stressed conditions (Takasaki et al., 2010; Song et al., 2011). Furthermore, in rice the up regulation of the *OsNAC5* stimulated the stress tolerance by increasing the levels of stress inducible rice genes like *LEA3* (Takasaki et al., 2010; Figure 2B). Moreover, *OsRAB16b* belongs to *LEA* genes that are expressed in response to abiotic stresses in both somatic and reproductive tissues (Tunnacliffe and Wise, 2007; Bies-Etheve et al., 2008). Broadly, *LEA* proteins encoded by the *LEA* genes render the property of acclimatization to the plants particularly under stressful conditions (Lenka et al., 2011).

In eukaryotes, the protein turnover is maintained by the Ubiquitin (Ub)-26S proteasome pathway. During the process of ubiquitination, the target proteins are linked to multiple Ub chains by ubiquitin ligases such as E1, E2, and E3 (Kraft et al., 2005; Stone et al., 2005). According to previous reports, the RING E3 Ub ligases play a vital role particularly in response to drought stress in rice (Bae et al., 2011; Ning et al., 2011; Park et al., 2011). Till date, five homologs of *OsRDCP* were identified in rice which possesses a single *RING* motif in their N-terminal regions (Khattab et al., 2014). The *OsRDCP1* is one among the five homologs which combated the dehydration stress in rice was up-regulated by the application of Si (Bae et al., 2011; Khattab et al., 2014; Figure 2C). Similarly, choline mono oxygenase the product of *OsCMO* is a primary enzyme involved in the biosynthesis of glycine betaine (Burnet et al., 1995). The glycine betaine is widely known for its osmolytic property that renders abiotic stress tolerance in several plants. Hence, the Si-mediated enhancement of *OsCMO* gene levels improved the stress tolerance in rice (Burnet et al., 1995; Figure 2D). The silicon-dependent up-regulation of transcription factors could interact with the cis-elements located in the promoter region of genes involved in stress resistance and trigger the stress tolerance against abiotic and biotic stresses. These regulatory genes might also induce the transcription of genes associated with the defense related or stress responsive pathways such as phenylpropanoid pathway, ABA-dependent or ABA-independent regulatory pathways to protect the plants from stress.

## Modulation of genes involved in water uptake and transportation upon Si nutrition

**Figure 3:**
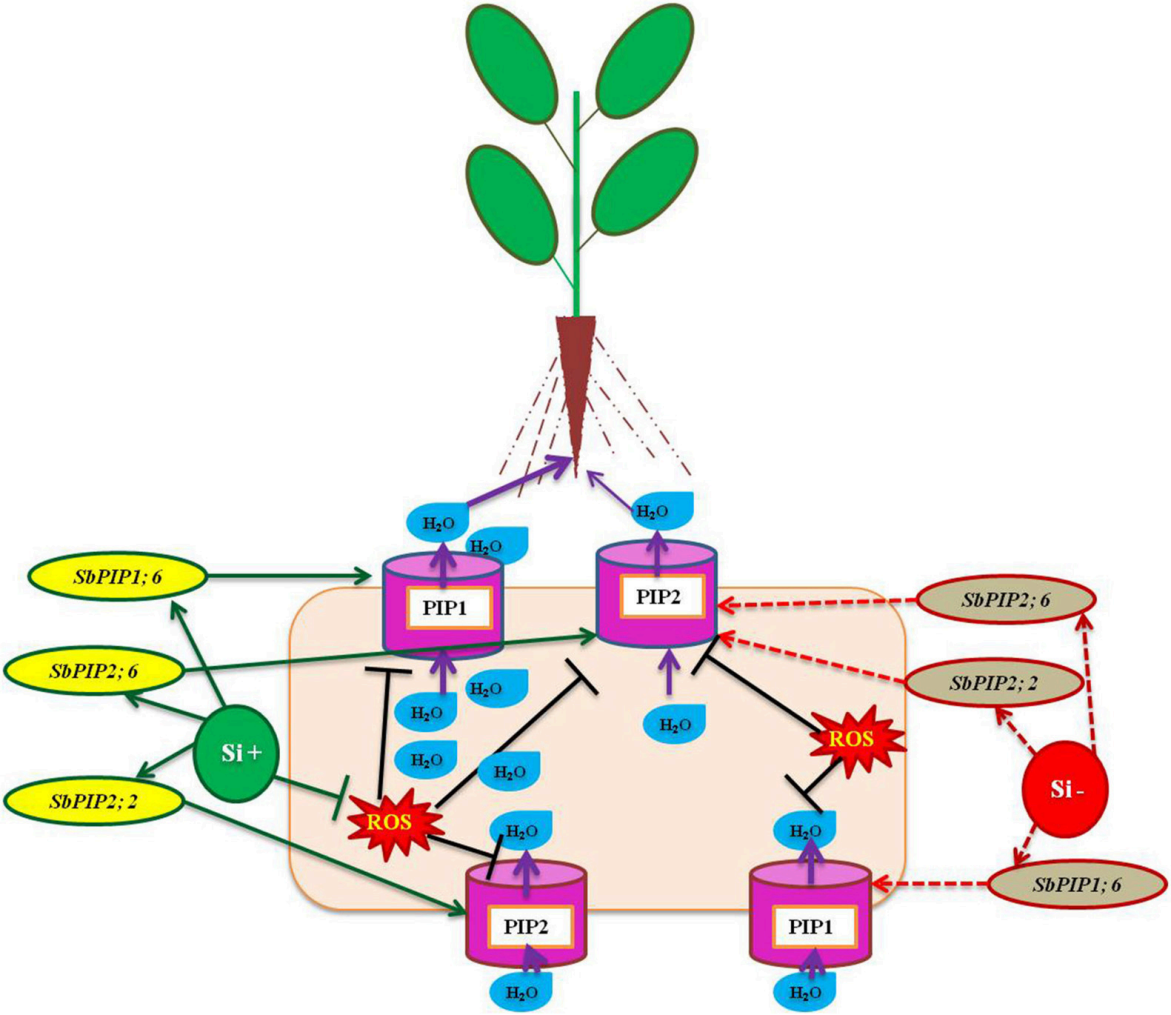
A model representation of aquaporin related genes regulation under osmotic stress condition with or without Si supplementation. The green standard arrows represent the up-regulation of genes in Si+ and red dotted arrows indicate the down-regulation of genes and corresponding functions upon stress in Si- conditions. The down regulation of PIP genes could result in the lesser activity of Aquaporin mediated transportation however upon Si augmentation the up-regulation of PIP genes improve the water status of the plants under stress. *SbPIP1*, Sorghum plasma membrane intrinsic protein, and PIP (plasma membrane intrinsic protein). The model was conceived based on the interpretation from the following literatures (Liu et al., 2015; Maurel et al., 2015).

Aquaporins are essential transmembrane proteins that maintain the uptake and movement of water molecules across cell membranes, particularly under abiotic stress condition (Boursiac et al., 2005). However, the function of aquaporin has been implicated by several factors such as abscisic acid, level of calcium ions, free radicals, and ethylene (Azad et al., 2004; Parent et al., 2009; Hu et al., 2012). According to Boursiac et al. (2008) the activity of aquaporin is susceptible to a mere change in the level of ROS, for instance the H_2_O_2_ stimulated by salt stress resulted in the prevention of aquaporin function by modulating the oxidant gating, phosphorylation condition, and re-localization of aquaporin. The amendment of Si enhanced the uptake of water particularly under salinity stress in several plants however the molecular mechanism behind the improvement of water status is unclear until recently. In *Sorghum bicolor*, the application of Si enhanced water uptake by increasing the activity of aquaporin by the up-regulation of *SbPIP1;6, SbPIP2;2*, and *SbPIP2;6* encoding plasma membrane intrinsic protein (PIP), the copious aquaporin in root (Liu et al., 2015; Figure 3). In addition, the higher expression of genes related to aquaporin results in the rapid water uptake which also dilutes the excess concentration of Na^+^ ions lethal for the plants (Gao et al., 2010). In accordance with the existing reports on the uses of aquaporin up regulation, the findings of Sutka et al. (2011) illustrated that the abundance of aquaporin genes in the roots balance the water uptake by the plants even under water-deficit conditions. In both normal and stressful environment the regulation of aquaporins plays a vital role in maintaining the proper uptake and transportation of water and solutes in plants. The enhancement of aquaporin related genes by silicon might substantiate the improvement of water status in plants treated with Si in salinity and drought stressed plants. The improvement of water status and ion balance aid in the reclamation of plants from stress.

## Regulation of polyamine biosynthesis genes by Si supplementation

**Figure 4:**
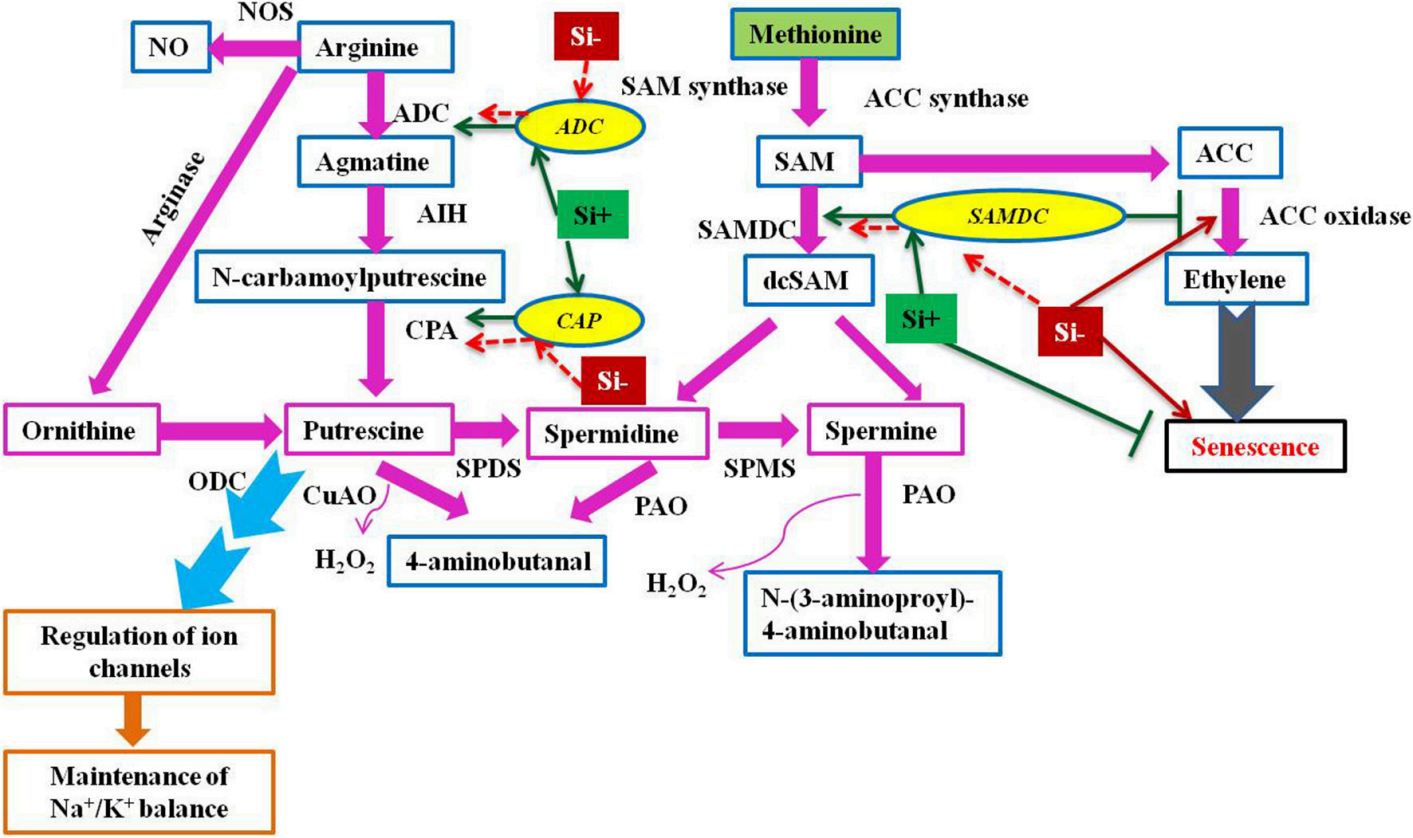
A schematic illustration of polyamine biosynthesis gene regulation under stress condition with or without Si supplementation. The green standard arrows represent the up-regulation of genes in Si+ and red dotted arrows indicate the down-regulation of genes and corresponding functions upon stress in Si- conditions. *SAMDC*, S-adenosyl-L-methionine decarboxylase; *ADC*, arginine decarboxylase; *CAP*, N-carbamoylputrescine amidohydrolase; ACC, 1-aminocyclopropane- 1-carboxylic acid; SAM, S-adenosyl-L-methionine; ODC, ornithine decarboxylase; SPDS, spermidine synthase; SPMS, spermine synthase; CuAO, copper amine oxidase; PAO, polyamine oxidase; AIH, agmatine iminohydrolase; PAO, polyamine oxidase; NOS, nitric oxide synthase. The model was conceived based on the interpretation from the following literatures (Mizoi et al., 2012; Khattab et al., 2014; Shi and Chan, 2014; Kurepin et al., 2015; and Yin et al., 2016).

Plants with higher levels of polyamines like putrescine, spermidine, and spermine reported to possess more resistance against environmental onslaughts like salinity (Liu et al., 2006; Chai et al., 2010; Quinet et al., 2010; Pottosin and Shabala, 2014). Furthermore, the elevated levels of genes responsible for the synthesis of polyamines mitigates the negative effects of oxidative stress (Roy and Wu, 2001; Tang et al., 2007). Therefore, the role of polyamines in stress resistance is becoming inevitable and the molecular insight into the Si dependent modulation of polyamines has been reported in *Sorghum bicolor* (Yin et al., 2016). The augmentation of Si elevated the expression level of S-adenosyl-L-methionine decarboxylase (*SAMDC*) gene which encodes a vital enzyme involved in the biosynthesis of polyamines. In addition, the report also hypothesized that the Si-mediated salt tolerance in sorghum has been associated with the polyamines and ethylene synthesis. On the contrary to *SAMDC*, the Si application retarded the synthesis of ethylene in sorghum under salinity stress. Since the polyamines such as spermidine and spermine share a common precursor, S-adenosyl-L-methionine (SAM) with ethylene, it is considered as the presence of a competitive environment amongst the polyamines and ethylene (Pandey et al., 2000). Therefore, in order to reduce the competitive condition, Si could have reduced the ethylene level by the inhibition, 1-aminocyclopropane-1-1-carboxylic acid (ACC) an important ethylene precursor (Yin et al., 2016). The supplementation of Si balanced the metabolism of polyamines and ethylene to mitigate abiotic stress (Figure 4). Polyamines are involved in various vital processes such as replication, transcription and translation, stabilization of membranes, and modulation of enzyme activities in addition to stress tolerance. Hence, the regulation of polyamine biosynthesis genes by Si could not only helps in the stress alleviation but also improves the fundamental processes in cells upon stress and increase the growth and development of plants.

## Silicon-Mediated expression of defense responsive genes

**Figure 5:**
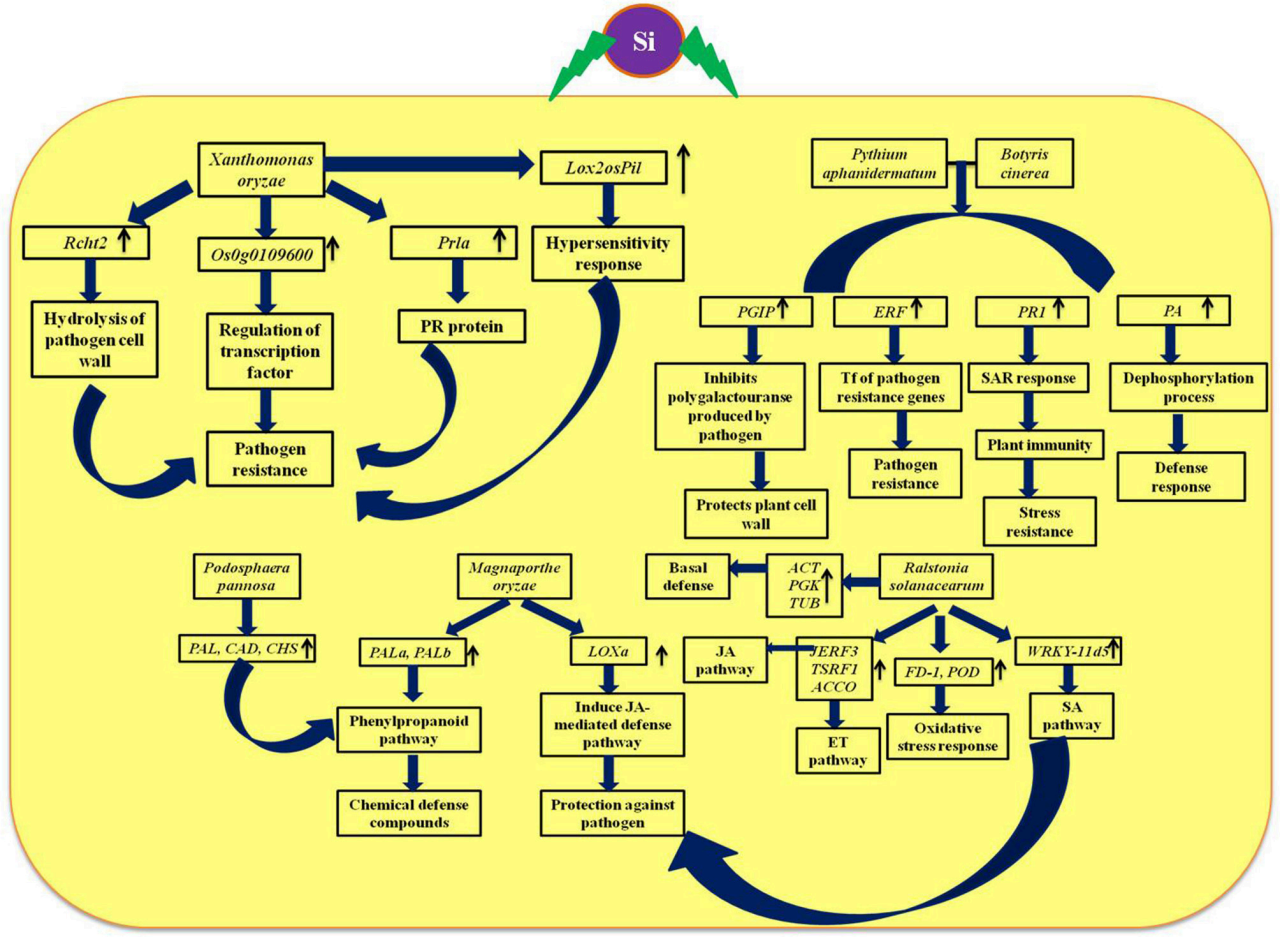
Schematic representation of Si-mediated regulation of vital genes associated with defense and phytohormones upon biotic stress. *Rcht2*, Chitinase; *Prla*, PR-1; *Lox*, Lipoxygenase; *PAL*, phenylalanine ammonia lyase; *CAD*, cinnamyl alcohol dehydrogenase; *CHS*, Chalcone synthase; *PGIP*, Polygalactouranase inhibitor protein; *PA*, phosphatase associated to defense; *PR-1*, pathogenesis-related protein; *ERF*, Ethylene response factor; *JERF*, Jasmonate and ethylene responsive factor 3; *TSRF*, Tomato stress-responsive factor; *ACCO*, 1-aminocyclopropane-1-carboxylate oxidase; *FD-1*, Ferredoxin-I; *POD*, Peroxidase; *WRKY II*, WRKY group II transcription factor; SA, Salicylic acid; JA, Jasmonic acid. The diagram was conceived based on the interpretation from the following literatures (Ghareeb et al., 2011b; Shetty et al., 2011; Rahman et al., 2015; El-Garhy et al., 2016; Song et al., 2016).

The defensive role of Si against biotic and abiotic stresses has been evidenced by several plant biologists. Especially, the Si-mediated protection against potential plant diseases such as powdery mildew and rice blast disease has been studied widely (Figure 5). The extensive study by Rodrigues et al. (2004) elucidated the positive regulation of genes related to the defense mechanism such as chalcone synthase (CHS), phenylalanine-ammonia lyase (PAL), pathogenesis related protein (PR1), peroxidase (POX), chitinases, and β-1, 3-glucanases by Si upon *Magnaporthe grisea* infection. Among the listed genes, CHS is a rate limiting enzyme in the flavonoid biosynthesis pathway and PAL plays a vital role in the synthesis of secondary metabolites with potential chemical defense property via phenylpropanoid pathway (Rodrigues et al., 2004). Furthermore, the peroxidases enzymes are important for lignin biosynthesis which acts as the potential mechanical barrier against pathogens (Rhodes, 1994). Similarly, the pathogenesis related (PR-1) protein in combination with genes related to secondary metabolism acts as the primary outcome of the plant defense response (Zeier et al., 2004). Moreover, the supplementation of Si altered the expression pattern of defense genes in rice to render resistance against *Magnaporthe oryzae* (Brunings et al., 2009). In addition, Si application in rice also induced differential expression of heavy metal transport and detoxification related genes to mitigate the heavy metal toxicity (Brunings et al., 2009). Altogether, Si regulates the genes responsible for vital physiological functions in plants particularly under stressed environment. Among the several mechanism of stress tolerance reported to exhibit by silicon, instigation of defense mechanism is considered as the pivotal one. Particularly, Si-mediated induction of cascade of reactions via phenypropanoid pathway is responsible for the synthesis and accumulation of chemical defense molecules such as phenols, and flavanoids against pathogens. Apart from the phenypropanoid pathway, Si can also induce the systemic acquired resistance (SAR) by the regulation of genes involved in hypersensitivity response and jasmonic acid mediated defense pathway to protect against pathogen attack.

## Conclusions

Silicon is the modest and a major element of soil with enormous benefits to plants especially in the mitigation of abiotic and biotic stress. Owing to its numerous advantages, the inclusion of Si in modern cultivation systems likes soil-less cultivation system has been blooming in several areas. In recent days, the modernization of technology allows us to investigate the molecular level regulation of compounds which has been extended to study the role of silicon in gene level by plant biologists under different stress conditions. Even though, the research on understanding of molecular rationale behind the Si-mediated stress tolerance is in rudimentary stage, upcoming outcomes from the recent studies have shed light into several possible ways of Si-dependent stress tolerance in plants. Based on the current reports it is evident that silicon possess multifaceted role in the regulation of genes involved in photosynthesis, secondary metabolism, polyamine biosynthesis, transcription, and water uptake. The molecular level modulations triggered by Si supplementation under stressed environment corresponded to the physiological improvement of plant growth and recovery from stress. In addition, several other novel molecular mechanisms behind the stress alleviation by Si have to be unraveled in the future.

## Author contributions

AM, collected the literatures and wrote the manuscript; YA proof-read, finalized, and approved the manuscript.

### Conflict of interest statement

The authors declare that the research was conducted in the absence of any commercial or financial relationships that could be construed as a potential conflict of interest.
