# Supplementary material for: Silicon Regulates Potential Genes Involved in Major Physiological Processes in Plants to Combat Stress
Source: Front Plant Sci. 2017 Aug 3;8:1346. doi: 10.3389/fpls.2017.01346 (PMC5541085; doi:10.3389/fpls.2017.01346)
Supplement: Supplementary file 1 [file Table1.DOCX]

**Supplementary Table 1**. Advantages of silicon nutrition in different plants under abiotic and biotic stress conditions.

| **S. No.** | **Stress** | **Plant** | **Benefits of Si amendment** | **Reference** |
| --- | --- | --- | --- | --- |
| 1 | Chromium toxicity | *Brassica juncea* | Retardation of metal transport, improvement of photosynthesis. | Ashfaque, et al. 2017 |
| 2 | Drought | *Nicotiana rustica* | Enhancement of water status, photosynthesis, and antioxidant defense. | Hajiboland, et al., 2017 |
| 3 | Cadmium toxicity | Brassica campestris | Induction of antioxidant defense. | Wu, et al., 2017a |
| 4 | High temperature | *Fragaria X annanasa* | Maintenance of proteins related to photosynthesis and stress response. | Muneer et al., 2017 |
| 5 | Hyperhydricity | *Dianthus caryophyllus* | Protection of stomata from damage, regulation of protein expression, and mitigation of oxidative stress. | Soundararajan et al., 2017a |
| 6 | Ultra violet-B radiation | Triticum aestivum | Nitric oxide mediated initiation of antioxidant defense. | Tripathi, et al., 2017 |
| 7 | Acid rain stress | Oryza sativa | Improvement of root growth, reduction of H_2_O_2_, maintenance of mineral elements. | Ju et al., 2017 |
| 8 | Powdery mildew | Cucurbita pepo | Reduced the disease severity and incidence. | Vercelli et al., 2017 |
| 9 | Salinity stress | Oryza sativa | Alleviated salt stress by the regulation of jasmonic acid and antioxidant enzymes. | Abdel-Haliem et al., 2017 |
| 10 | Herbivore attack | Oryza sativa | Antagonistic effects of nitrogen supplementation on Si amendments. | Wu et al., 2017b |
| 11 | Fungal disease | Oryza sativa | Application of Si along with rhizobacteria alleviated the severity of leaf scald, shielded the photosynthetic apparatus from damage. | Bueno et al., 2017 |
| 12 | Cadmium toxicity | Pisum sativum | Improvement of growth, cadmium retardation in roots, and detoxification of reactive oxygen species (ROS). | Rahman et al., 2017 |
| 13 | Salt stress | *Solanum lycopersicum* | Si decreased the uptake of Na^+^ ions. | Yunus and Zari, 2017 |
| 14 | Salt stress | Rosa hybrida | Improvement of growth, protein expression, and regulation of redox homeostasis. | Soundararajan et al., 2017b |
| 15 | Salt stress | Abelmoschus esculentus | Si increases growth and reduces the negative effects of salinity. | Qadir et al., 2017 |
| 16 | Drought | Triticum aestivum | Si increased the expression of genes related to antioxidant enzymes and enhanced the contents of total phenols and flavonoids. | Ma et al., 2016 |
| 17 | Autotoxicity | Cucumis sativus | Si improved the germination and reduced the lipid peroxidation in plants affected by autotoxic agent. | Bu et al., 2016 |
| 18 | Alkaline stress | Zea maize | Si priming resulted in improvement of leaf water content, soluble sugars, proteins, free amino acids, and antioxidant enzyme activities. | Latef and Tran, 2016 |
| 19 | Aluminium toxicity | *Eucalyptus platyphylla* | Si reduced electrolyte leakage, malondialdehyde content, and H_2_O_2_ level. It increase pigmentation and gas exchange parameters. | Lima et al., 2016 |
| 20 | Cadmium toxicity | Triticum turgidum | Si decreased the cadmium uptake and armored the root surface from damage. | Rizwan et al., 2016 |
| 21 | Flooding | Hordeum vulgare | Si enhanced the growth process and reduced the oxidative destruction caused by flooding | Balakhnina et al., 2012 |

**References:**

Abdel-Haliem, M. E., Hegazy, H. S., Hassan, N. S., and Naguib, D. M. (2017). Effect of silica ions and nano silica on rice plants under salinity stress. Ecol. Eng*.* 99, 282–289. doi: 10.1016/j.ecoleng.2016.11.060

Ashfaque, F., Inam, A., Iqbal, S., and Sahay, S. (2017). Response of silicon on metal accumulation, photosynthetic inhibition and oxidative stress in chromium-induced mustard (*Brassica juncea* L.). South Afr. J. Bot*.* 111, 153–160. doi: 10.1016/j.sajb.2017.03.002

Balakhnina, T. I., Matichenkov, V. V., Wlodarczyk, T., Borkowska, A., Nosalewicz, M., and Fomina, I. R. (2012). Effects of silicon on growth processes and adaptive potential of barley plants under optimal soil watering and flooding. Plant Growth Regul*.* 67, 35–43. doi: 10.1007/s10725-012-9658-6

Bu, R., Xie, J., Yu, J., Liao, W., Xiao, X., Lv, J., et al. (2016). Autotoxicity in cucumber (*Cucumis sativus* L.) seedlings is alleviated by silicon through an increase in the activity of antioxidant enzymes and by mitigating lipid peroxidation. J. Plant Biol*.* 59, 247–259. doi: 10.1007/s12374-016-0526-1

Bueno, A. C. S. O., Castro, G. L. S., Silva Junior, D. D., Pinheiro, H. A., Filippi, M. C. C., and Silva, G. B. (2017). Response of photosynthesis and chlorophylla fluorescence in leaf scald-infected rice under influence of rhizobacteria and silicon fertilizer. Plant Pathol*.* doi: 10.1111/ppa.12690. [Epub ahead of print].

Hajiboland, R., Cheraghvareh, L., and Poschenrieder, C. (2017). Improvement of drought tolerance in tobacco (*Nicotiana rustica* L.) plants by silicon. J. Plant Nutr*.* doi: 10.1080/01904167.2017.1310887. [Epub ahead of print].

Ju, S., Yin, N., Wang, L., Zhang, C., and Wang, Y. (2017). Effects of silicon on *Oryza sativa* L. seedling roots under simulated acid rain stress. PLoS ONE 12:e0173378. doi: 10.1371/journal.pone.0173378

Latef, A. A. A., and Tran, L. S. P. (2016). Impacts of priming with silicon on the growth and tolerance of maize plants to alkaline stress. Front. Plant Sci*.* 7:243. doi: 10.3389/fpls.2016.00243

Lima, M. D. R., Barros, U. O. Jr., Barbosa, M. A. M., Segura, F. R., Silva, F. F., Batista, B. L., et al. (2016). Silicon mitigates oxidative stress and has positive effects in *Eucalyptus platyphylla* under aluminium toxicity. Plant Soil Environ. 62, 164–170. doi: 10.17221/85/2016-PSE

Ma, D., Sun, D., Wang, C., Qin, H., Ding, H., Li, Y., et al. (2016). Silicon application alleviates drought stress in wheat through transcriptional regulation of multiple antioxidant defense pathways. J. Plant Growth Regul*.* 35, 1–10. doi: 10.1007/s00344-015-9500-2

Muneer, S., Park, Y. G., Kim, S., and Jeong, B. R. (2017). Foliar or subirrigation silicon supply mitigates high temperature stress in strawberry by maintaining photosynthetic and stress-responsive proteins. *J. Plant Growth Regul.* doi: 10.1007/s00344-017-9687-5. [Epub ahead of print].

Qadir, A., Khan, S. A., Ahmad, R., Masood, S., Irshad, M., Kaleem, F., et al. (2017). Exogenous Ca_2_SiO_4_ enrichment reduces the leaf apoplastic Na^+^ and increases the growth of okra (*Abelmoschus esculentus* L.) under salt stress. Sci. Hortic*.* 214, 1–8. doi: 10.1016/j.scienta.2016.11.008

Rahman, M. F., Ghosal, A., Alam, M. F., and Kabir, A. H. (2017). Remediation of cadmium toxicity in field peas (*Pisum sativum* L.) through exogenous silicon. Ecotoxicol. Environ. Saf*.* 135, 165–172. doi: 10.1016/j.ecoenv.2016.09.019

Rizwan, M., Meunier, J. D., Davidian, J. C., Pokrovsky, O. S., Bovet, N., and Keller, C. (2016). Silicon alleviates Cd stress of wheat seedlings (*Triticum turgidum* L. cv. Claudio) grown in hydroponics. Environ. Sci. Pollut. Res. 23, 1414–1427. doi: 10.1007/s11356-015-5351-4

Soundararajan, P., Manivannan, A., Cho, Y. S., and Jeong, B. R. (2017a). Exogenous supplementation of silicon improved the recovery of hyperhydric shoots in *Dianthus caryophyllus* L. by stabilizing the physiology and protein expression. Front. Plant Sci*.* 8:738. doi: 10.3389/fpls.2017.00738

Soundararajan, P., Manivannan, A., Ko, C. H., and Jeong, B. R. (2017b). Silicon enhanced redox homeostasis and protein expression to mitigate the salinity stress in rosa hybrida ‘Rock Fire’. *J. Plant Growth Regul.* doi: 10.1016/j.pmpp.2011.02.003. [Epub ahead of print].

Tripathi, D. K., Singh, S., Singh, V. P., Prasad, S. M., Dubey, N. K., and Chauhan, D. K. (2017). Silicon nanoparticles more effectively alleviated UV-B stress than silicon in wheat (*Triticum aestivum*) seedlings. Plant Physiol. Biochem*.* 110, 70–81. doi: 10.1016/j.plaphy.2016.06.026

Vercelli, M., Minuto, A., Minuto, G., Contartese, V., Devecchi, M., and Larcher, F. (2017). The effects of innovative silicon applications on growth and powdery mildew control in soilless-grown cucumber (*Cucumissativus* L.) and zucchini (*Cucurbita pepo* L.). Acta Physiol. Plant*.* 39, 129. doi: 10.1007/s11738-017-2426-5

Wu, X., Yu, Y., Baerson, S. R., Song, Y., Liang, G., Ding, C., et al. (2017b). Interactions between nitrogen and silicon in rice and their effects on resistance toward the brown planthopper *Nilaparvata lugens*. Front. Plant Sci*.* 8:28. doi: 10.3389/fpls.2017.00028

Wu, Z., Liu, S., Zhao, J., Wang, F., Du, Y., Zou, S., et al. (2017a). Comparative responses to silicon and selenium in relation to antioxidant enzyme system and the glutathione-ascorbate cycle in flowering Chinese cabbage (*Brassica campestris* L. ssp. *chinensis* var. *utilis*) under cadmiumstress. Environ. Exp. Bot. 133, 1–11. doi: 10.1016/j.envexpbot.2016.09.005

Yunus, Q., and Zari, M. (2017). Effect of exogenous silicon on ion distribution of tomato plants under salt stress. Commun. Soil Sci. Plant Anal*.* doi: 10.1080/00103624.2017.1311908. [Epub ahead of print].
